# Observations on Twin Cases

**Published:** 1812-08

**Authors:** 


*To the Editors of the Medical and Physical Journal.*

GENTLEMEN,

MR. FOGO, in your Journal for May, has replied to some observations I made on his practice in twin cases, in No. 156. He appears greatly to have misunderstood me in some passages, and in others not to have fairly stated my arguments.

When I expressed myself desirous of farther authority than his mere *ipse dixit*, I neither doubted his sentiments nor suspected his veracity, but wished to be referred to any medical writer who had recommended the same practice. We are naturally inclined to prefer the advice given by the most eminent practitioners, to that of a solitary individual; still I am not so far prejudiced in favor of any particular doctrine, as to reject any *vis argumenti* merely because it is not in unison with general opinion; and, to convince Mr. F. I am open to conviction, and unwilling to condemn a practice which I have not hitherto given a trial, it is my intention in the next twin case that occurs to me, to adopt his practice; and, should the result be as favorable as he represents, the profession will be indebted to him for a very material improvement, and I shall consider my having been desirous of *farther authority* as a very fortunate circumstance.

When I mentioned a gentleman having waited *twelve* hours without any ill consequence ensuing, it was not with the intention of recommending the practice (nor do the London professors), but to prove it might be done without danger to the mother or child.

Mr. F. asks, "if I ever witnessed the strong and rapid contraction of the os uteri, and other parts, in the space of twelve hours?"—I have; and known the second child delivered by the efforts of nature in a quarter of an hour after the first, without the woman or attendants being sensible of it. This acknowledgment Mr. F. will perceive to be an argument in favor of his practice: I am happy it should be so, and and should feel more pleasure in conducing to any improvement in the profession, than in the indulgence of idle speculations which could not bear the touch-stone of experience.

Mr. F. " never witnessed syncope and irregular contraction of the uterus take place in consequence of the too sudden expulsion of the child," nor can I, he says, have any authority to prove the cause and effect. Having known syncope occur myself, and to others under similar circumstances, I imagined it was not an unusual occurrence. The cause has been variously accounted for by different writers; some suppose it originates from the pressure of the gravid uterus being too quickly removed from the larger vessels, in which the free circulation of the blood was before partially obstructed; the blood suddenly descends, which has the same effect as the sudden *loss* of a large quantity. We know a man may lose six or seven pounds gradually without experiencing any thing farther than mere debility, but half or even a much less quantity suddenly lost, from the violent derangement it would induce in the sanguiferous system, would bring on syncope, or even death. Another reason is the sudden descent or removal of the abdominal viscera. Every one is acquainted that in Paracentesis Abdominis the sudden removal of the pressure on the viscera and diaphragm will produce swooning, and other alarming symptoms, unless the water is drawn off very gradually, or pressure is made by bandage, in order that the support may not be too suddenly removed. These are the causes usually assigned; I submit it to the judgment of others to determine which is the most probable. The irregular contraction of the uterus from the too sudden expulsion of the child, is what might naturally be expected; if the child is expelled as it were *instantly*, the contraction is as likely (from there being no resistance) to take place at the cervix as the fundus, in lieu of gradually contracting from the latter as the child advances. This I imagine to be the cause; the effect is obvious.

When I advised opposing the passage of the child, I did not intend we should counteract the efforts of nature any farther than preventing its too sudden expulsion, and guarding against the laceration of the perinæum. When the contraction is particularly strong, and the parts are not well dilated, we are informed laceration is liable to take place. I said it had very rarely taken place, but if it *ever* has are we not bound to prevent it by taking every reasonable precaution? and, as I before observed, there can be no *harm* in the practice. I have a much higher opinion of the London practitioners than to imagine they consider guarding the the perinæum as the *summum bonum* of midwifery, nor can I believe Mr. F. seriously thinks so; was that the case, any lady's maid might indeed take the chair, but there are numerous difficulties liable to occur in which she would be very happy to resign it.

Mr. F. thinks my reasoning not very scientific, where I observed, "at the time of his delivering the second, the effusion of blood already taken place must have nearly filled the uterus, even supposing it to have not been partially contracted, &c." and asks, "whether this is the way of stopping hæmorrhage in London, to allow the uterus to be filled with blood for the sake of pressing on the mouths of the vessels?" I can assure him it is not, and plugging the vagina is never had recourse to, but as the *sine qua non*, when every other expedient our ingenuity could devise has failed. Mr. F. always considered plugging the vagina as a very unscientific method of suppressing or preventing uterine hæmorrhage, but, when other means fail, what are we to have recourse to? something must be done, and, that it has been successfully, we have only to refer to those authors who have written on the subject.

Do practitioners, says Mr. F. consider "how much blood a recently-emptied torpid uterus will contain before the blood can sufficiently press on the mouths of the vessels?" There is not a more difficult problem in Euclid to solve than this question, as the capacity of the uterus in different subjects must make a very material difference. It being a twin case that I alluded to, I think the quantity effused nearly, if not fully, equal to the weight of the child.

Mr. F. has been told, the doctrine of leaving every thing to Nature originated in Dr. Hunter, who, he heard, had occasion to change his practice. I suppose he alludes to the doctor's always leaving the patient as soon as the child was born, and trusting the care of the placenta to the nurse (in one instance hæmorrhage ensued, and the lady died); but this is not the London practice at present.

Mr. F. asks, "does the surgeon leave fracture, dislocation, &c. to Nature? there seems nothing in the various branches left to nature but that of midwifery;" this is surely *totâ viâ, toto cœlo errare.* I cannot perceive the least similitude between the cases: the former are *unnatural* effects, in which we cannot interfere too soon; the latter is a *natural* effort of Nature, in which, generally speaking, the less we interfere the better. There is a contradiction at the latter part of Mr. F.'s letter: he admits that midwifery requires in *many* cases as much attention as some other branches of the profession, and at the same time proposes leaving the care of it it to be undertaken by any discreet, humane, illiterate, old woman. I never was acquainted with any of the good women in Northumberland; but, if they are not rather more scientific than those in Kent, I think the obstetric art may as safely remain in the hands of the present medical practitioners; and, in regard to the imposition of great fees being paid to a certain description of men, I cannot agree with Mr. F. as I always consider the fee very inadequate to the trouble and anxiety we frequently experience.

Nothing is more likely to conduce to the improvement of the profession than argument divested of personal reflections. The practice of Mr. F. is certainly novel; but, till experience shall have proved whether the success of other gentlemen is equal to his, we must suspend our judgment. Should any of your readers feel inclined to make the experiment, I hope they will favor us at some future period with the impartial result.

I am, Gentlemen,

Your very humble Servant,

*Hythe, May* 24, 1812. T. E. B.

P. S. Your correspondent A. B. in No. 159, wishes to be informed if there is any authentic history of the tree that produces the caoutchouc, with the mode in which the Indians prepare it. He will find an account of the latter in Quincy's Lexicon Medicum, edited by Dr. Hooper, under the head of *Siphonia elastica;* in Murray's Chemistry; as also in Chaptal's Chemistry, where there is a concise account of the tree, &c. but for more accurate details he refers you to an account in the Acad. des Sciences, 1751, by Mr. Condamine.

